# Omega-3 intake is associated with liver disease protection

**DOI:** 10.3389/fpubh.2023.1192099

**Published:** 2023-07-19

**Authors:** Mara Sophie Vell, Kate Townsend Creasy, Eleonora Scorletti, Katharina Sophie Seeling, Leonida Hehl, Miriam Daphne Rendel, Kai Markus Schneider, Carolin Victoria Schneider

**Affiliations:** ^1^Department of Medicine III, Gastroenterology, Metabolic Diseases and Intensive Care, University Hospital RWTH Aachen, Aachen, Germany; ^2^Department of Biobehavioral Health Sciences, School of Nursing, University of Pennsylvania, Philadelphia, PA, United States; ^3^Department of Genetics, Perelman School of Medicine, University of Pennsylvania, Philadelphia, PA, United States; ^4^The Institute for Translational Medicine and Therapeutics, The Perelman School of Medicine, University of Pennsylvania, Philadelphia, PA, United States

**Keywords:** Omega-3 fatty acids, NAFLD, liver disease, primary prevention, alcoholic liver disease (ALD)

## Abstract

**Background:**

Non-alcoholic fatty liver disease (NAFLD) and alcoholic liver disease are among the most common liver diseases worldwide, and there are currently no Food and Drug Administration (FDA)-approved treatments. Recent studies have focused on lifestyle changes to prevent and treat NAFLD. Omega-3 supplementation is associated with improved outcomes in patients with chronic liver disease. However, it is unclear whether Omega-3 supplementation can prevent the development of liver disease, particularly in individuals at an increased (genetic) risk.

**Methods:**

In this UK Biobank cohort study, we established a multivariate cox proportional hazards model for the risk of incident liver disease during an 11 year follow up time. We adjusted the model for diabetes, prevalent cardiovascular disorders, socioeconomic status, diet, alcohol consumption, physical activity, medication intake (insulin, biguanides, statins and aspirin), and baseline characteristics.

**Results:**

Omega-3 supplementation reduced the risk of incident liver disease (HR = 0.716; 95% CI: 0.639, 0.802; *p* = 7.6 × 10^−9^). This protective association was particularly evident for alcoholic liver disease (HR = 0.559; 95% CI: 0.347, 0.833; *p* = 4.3 × 10^−3^), liver failure (HR = 0.548; 95% CI: 0.343, 0.875; *p* = 1.2 × 10^−2^), and non-alcoholic liver disease (HR = 0.784; 95% CI: 0.650, 0.944; *p* = 1.0 × 10^−2^). Interestingly, we were able to replicate the association with reduced risk of NAFLD in a subset with liver MRIs (HR = 0.846; 95% CI: 0.777, 0.921; *p* = 1.1 × 10^−4^). In particular, women benefited from Omega-3 supplementation as well as heterozygous allele carriers of the liver-damaging variant PNPLA3 rs738409.

**Conclusions:**

Omega-3 supplementation may reduce the incidence of liver disease. Our study highlights the potential of personalized treatment strategies for individuals at risk of metabolic liver disease. Further evaluation in clinical trials is warranted before Omega-3 can be recommended for the prevention of liver disease.

## Introduction

Non-alcoholic fatty liver disease (NAFLD) and alcoholic liver disease are two of the most prevalent liver diseases worldwide, affecting millions of people across the globe (1–3). Despite their high prevalence and potential for serious complications, there are currently no Food and Drug Administration (FDA)-approved treatments for either condition (4). Recent research has focused on the potential benefits of lifestyle modifications, including targeted dietary interventions and supplements, for their prevention and treatment (5–8). Among the lifestyle modifications studied for their potential benefits in treating NAFLD and alcoholic liver disease are Omega-3 fatty acids, including eicosapentaenoic acid (EPA), docosapentaenoic acid (DPA), and docosahexaenoic acid (DHA) (9). Omega-3 fatty acids have been shown to have a variety of health benefits, including reduced inflammation and improved cardiovascular health. Recent studies have shown that supplementation with DHA can improve liver and visceral fat in children with NAFLD, and supplementation with DHA and EPA can reduce liver fat (5, 6). However, it is unknown if Omega-3 supplementation has a preventive effect against the development of liver disease in the general population. Given the increasing prevalence of liver disease and the lack of FDA-approved treatments, it is compelling to investigate the potential benefits of Omega-3 fatty acid supplementation for the prevention of liver disease. Therefore, we used the UK Biobank to investigate the effect of Omega-3 fatty acid supplementation on the incidence of liver disease in a large population-based cohort.

## Methods

### UK biobank

For our study, we used the UK Biobank Resource with application number 71300. The study was approved by the Northwest Multicenter Research Ethics Committee and conducted in accordance with the Declaration of Helsinki and Istanbul after written informed consent was obtained. The UK Biobank (UKB) is a multicenter research study with 22 participating centers. Enrolment studies were conducted between 2006 and 2010. The respective date was set as baseline. Death or end of data collection in May 2021 was defined as the end of follow-up. After informed consent was obtained for clinical data collection and genotyping, a total of 502,511 individuals aged 37 to 73 years were enrolled in the study. The diagnoses included in our study were coded using the ‘International Classification of Diseases and Related Health Problem' (ICD-10). The latter were assigned to the patients and continuously updated via the hospital admission code. Death data were collected in the UKB via the National Death Register, including the leading diagnosis of death and the date of death.

Briefly, Omega-3 supplementation was tracked in a questionnaire and validated by lipidomic data, which were measured in 96,701 individuals by nuclear magnetic resonance. We included participants without any baseline diagnosis of liver disease (K70-K77, C22.0) and performed an analysis of reported Omega-3 intake with new diagnosis of liver disease over the 11-year study period. Detailed information can be found in Supplementary material (Supplementary Table S1).

### Exclusion criteria and missing data

**Figure 1:**
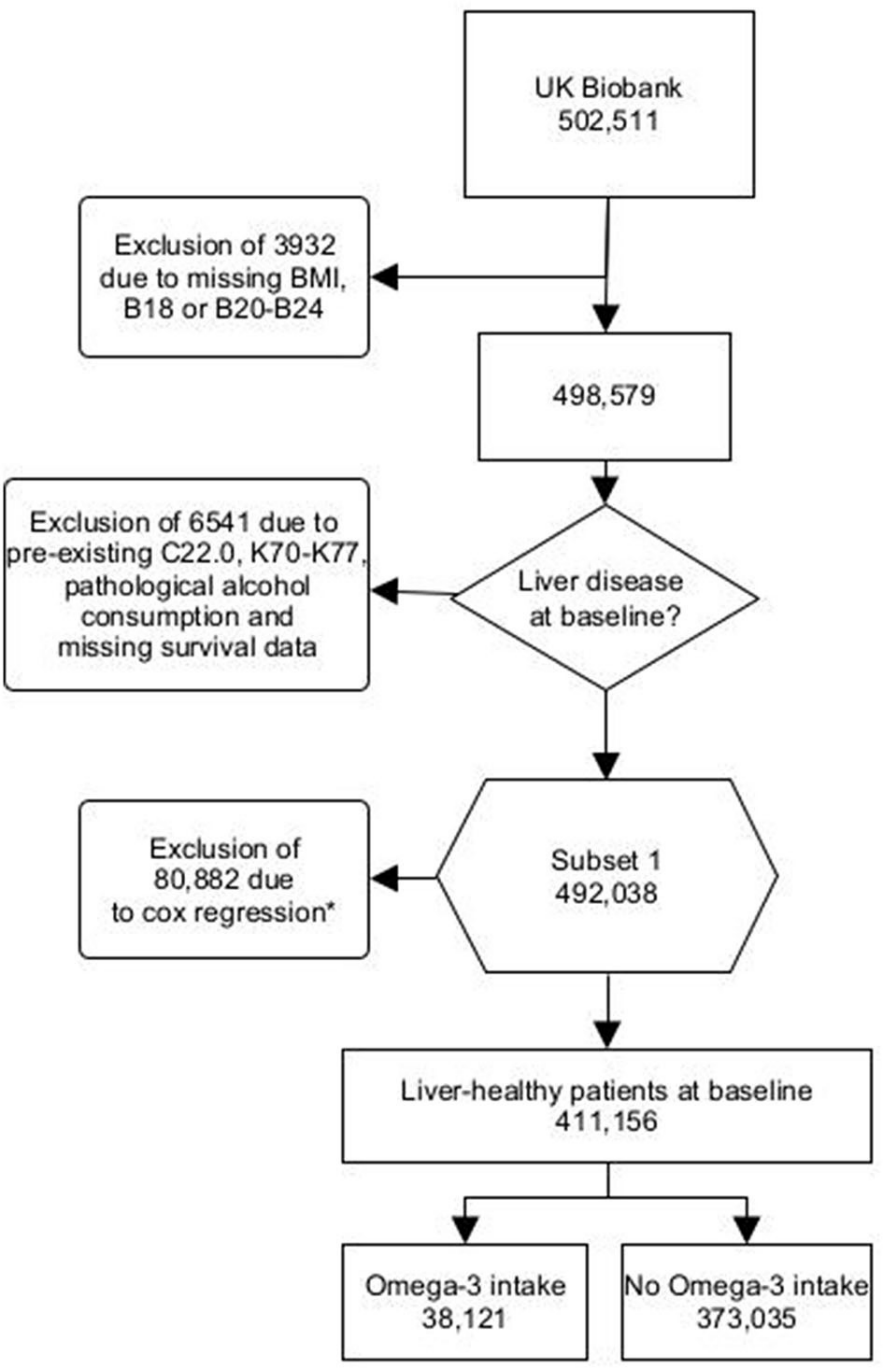
Flowchart UK Biobank Omega-3 users - B18, Chronic viral hepatitis; B20, Human immunodeficiency virus disease resulting in infectious and parasitic disease; B21, Human immunodeficiency virus disease resulting in malignant neoplasms; B22, Human immunodeficiency virus disease resulting in other specified diseases; B23, Human immunodeficiency virus disease resulting in other conditions; B24, Unspecified human immunodeficiency virus disease; C22.0, Hepatocellular carcinoma; K70, Alcoholic liver disease; K71, Toxic liver disease; K72, Hepatic failure, not elsewhere classified; K73, Chronic hepatitis, not elsewhere classified; K74, Fibrosis and cirrhosis of liver; K75, Other inflammatory liver diseases; K76, Other diseases of liver; K77, Liver disorders in diseases classified elsewhere. *Missing values were excluded from the cox proportional hazards model. This was the case for physical activity, diet, alcohol consumption and socioeconomic status.

We excluded individuals with any diagnosis of pre-existing liver diseases (K70-K77, C22.0), HIV infection (B20-B24), chronic hepatitis (B18) or missing body mass index (BMI) data (Figure 1). We also excluded individuals with pathological alcohol consumption (alcohol consumption >60 g/day for men and >40 g/d for women) (10). Individuals who consumed alcohol below the pathological cut-off were not excluded. In addition, we excluded one patient due to missing survival data (Figure 1).

Furthermore, patients were excluded from the multivariate cox proportional hazards model. Patients who reported being unable to walk (response option: “unable to walk:) were considered as “0 days/week walked”. Missing data for vegetable, fruit, fish and meat intakes were excluded. Likewise, missing data for alcohol consumption (in g/d) and socioeconomic status (Townsend Index) were excluded (Figure 1).

### Omega-3 intake

Omega-3 intake was recorded using a numerical code (Supplementary Table S1). We used data from the UKB that included people with regular supplementation of Omega-3 defined as daily, weekly or monthly Omega-3 intake. Non-users did not consume any supplemental Omega-3 fatty acids. Moreover, we used the plasma metabolic profile data available in the UKB as intake control.

### Metabolomics

**Figure 2:**
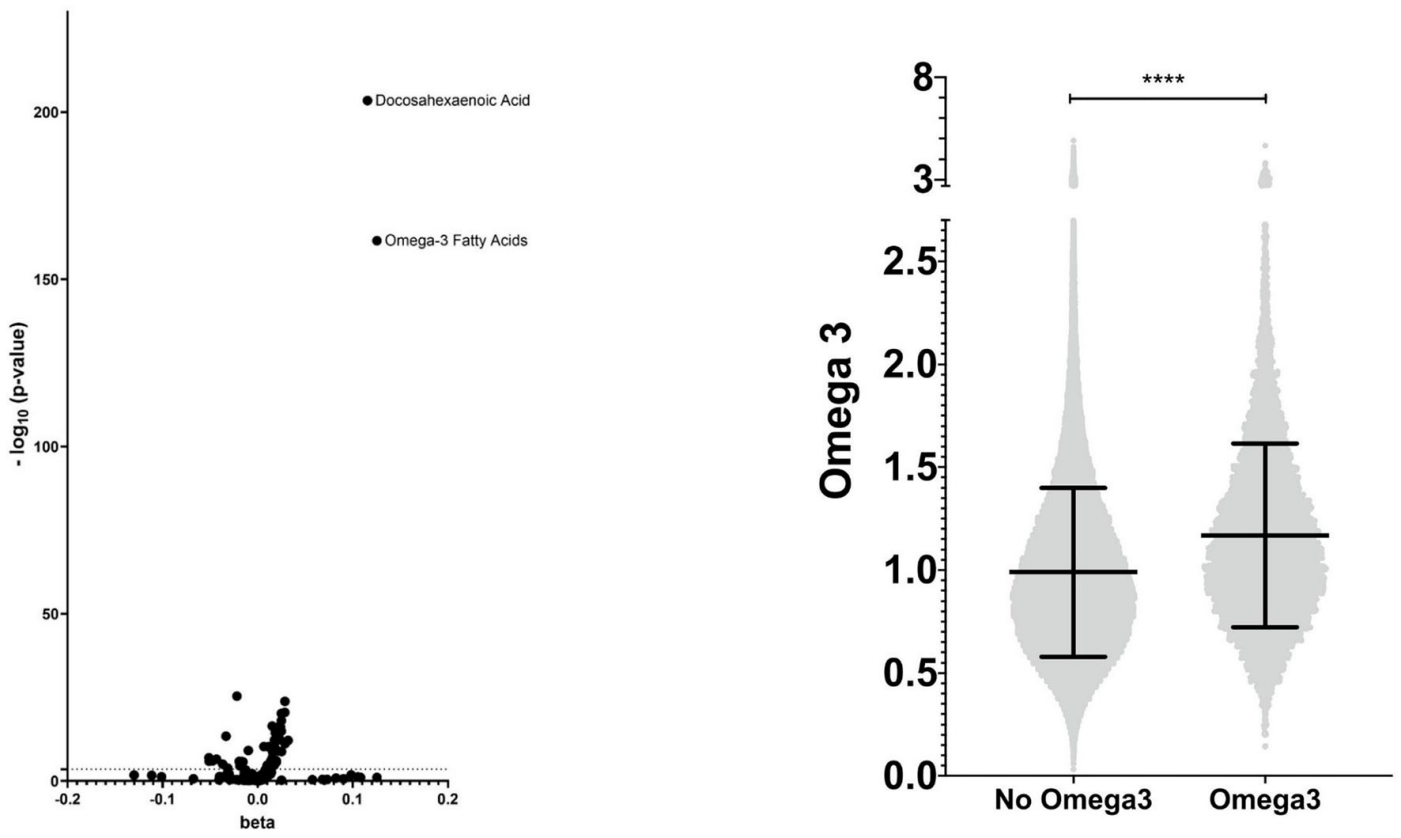
Metabolic profile - visualization of the lipidomic profile of Omega-3 users compared with non-users and distribution of Omega-3 serum levels. **** means <0.0001.

Using nuclear magnetic resonance, the UKB determined the metabolic profiles of 105,348 participants, of whom 96,701 individuals were included in our study. 90,965 had a measurement of Omega-3 serum level. We compared beta between individuals taking Omega-3 and those without intake to compare metabolites. The Bonferroni correction was performed to avoid type I error due to multiple testing. Values -log_10_(p/59) >3.1 were considered significant (Supplementary Table S2, Figure 2). We further examined the distribution of Omega-3 intake in patients with lipidomic data and additionally repeated the analyses (Supplementary Table S3).

### MRI confirmed steatosis

In UKB 40,797 individuals underwent liver MRIs to determine proton density fat fractions. Steatosis was defined as a liver fat fraction of >5% (11). A total of 31,216 individuals who received liver MRIs were included in the study.

### Genetic disposition

The UKB provided genetic analyses of 488,377 individuals. We investigated carriers of known liver-associated variants. Variants examined were patatin-like phospholipase domain-containing protein 3 (*PNPLA3*) rs738409, transmembrane 6 superfamily member 2 (*TM6SF2*) rs58542926, hydroxysteroid 17-beta dehydrogenase 13 (*HSD17B13*) rs72613567, and mitochondrial amidoxime reducing component 1 (*MTARC1*) rs2642438. We examined wildtype, heterozygous, and homozygous minor allele carriers.

### Primary and secondary outcomes

The primary outcome was the development of incident liver disease (K70-K77) or a new diagnosis of hepatocellular carcinoma (C22.0) after baseline. As a secondary outcome, we replicated the results using multivitamin supplements, vitamin C, or vitamin B12.

### Multivariate cox proportional hazards model

Covariates included in the multivariate cox proportional hazards model were age, sex, BMI, ethnicity, diabetes mellitus, hypertension, ischaemic heart disease, dyslipidemia, number of medications taken, as well as intake of insulin, biguanides, statins and aspirin. In addition, we integrated physical activity with “Number of days/week walked”, “Number of days/week of moderate physical activity”, and “Number of days of vigorous physical actitivity”. We further included nutritional factors with the help of daily vegetable and fruit consumption, and weekly consumption of fish and meat. In addition, alcohol consumption (g/d) was included as a covariate to account for alcohol consumption below the pathological cut-off. We determined socioeconomic status using the Townsend Index as a covariate (12).

### Statistical analysis

In the multivariate cox proportional hazards model, we considered influencing confounders as covariates. We included the covariates: Age, sex, BMI, ethnicity, diabetes mellitus, hypertension, ischemic heart disease, dyslipidemia, number of medications taken, insulin, biguanides, statins, aspirin, “number of days/week walked”, “number of days/week of moderate activity”, “number of days/week of vigorous activity”, daily fruit and vegetable consumption, weekly fish and meat consumption, daily alcohol consumption in g/d, and the Townsend index as a socioeconomic factor. Results are expressed as mean ± standard deviation (±SD). The 95% confidence intervals (CI) are given in parentheses for the hazard ratios (HR). Statistical significance was set at *p*-value < 0.05. Statistical analyses and graphical visualization were performed using R version 4.1.2 (R Foundation for Statistical Computing; Vienna, Austria), SPSS Statistics version 27 (IBM; Armonk, NY, USA), Prism version 8.0.1 (GraphPad, LaJolla, CA, USA), and yEd Graph Editor version 3.21.1.

## Results

### Omega-3 is associated with risk reduction for incident liver disease

A total of 411,156 individuals were included, 373,035 participants without intake of Omega-3 (non-users) and 38,121 with regular (self-reported as daily, weekly, or monthly) intake of Omega-3 (Omega-3 users) (Figure 1, Supplementary Table S2). First, we validated the reported Omega-3 intake by using lipidomic data (Figure 2). Indeed, Omega-3 users had significantly increased levels of Omega-3 (+19.0%), compared to non-users (Figure 2, Supplementary Table S2).

Table 1 provides a comparison of the basic characteristics between two groups: those with no Omega-3 intake and those with Omega-3 intake. Interestingly, patients with Omega-3 took more medications at baseline, but were more active, ate more vegetables and fish, and came from a socioeconomically advantageous background (Table 1).

**Table 1:** Comparison of the basic characteristics.

	**No Omega-3 intake**	**Omega-3 intake**	***p*-value**
	**(*N* = 373,035)**	**(*N* = 38,121)**	
Age (Years)	56.18 ± 8.13	58.99 ± 7.14	9.9e-300
Sex (% Women)	54.4	59.7	2.44e-87
BMI (kg/m^2^)	27.33 ± 4.75	27.01 ± 4.43	5.04e-42
Ethnicity (% White)	94.8	97.3	6.68e-102
Number of medications	2.36 ± 2.81	5.29 ± 3.10	9.9e-300
Aspirin intake	13.1	16.7	1.18e-82
Statin intake	15.7	17.1	2.64e-12
Insulin intake	1.0	0.7	3.78e-08
Biguanide intake	2.7	2.1	9.82e-14
Diabetes mellitus type II (E11)	5.3	4.5	2.12e-12
Arterial hypertension (I10)	21.2	22.5	1.78e-09
Angina pectoris (I20)	4.7	4.8	0.24
Chronic ischaemic heart disease (I25)	6.8	7.2	3.0e-03
Disorders of lipoprotein metabolism and other lipidemias (E78)	10.0	10.8	4.0e-06
Number of days/week walked	5.40 ± 1.94	5.51 ± 1.86	3.60e-28
Number of days/week of moderate physical activity	3.63 ± 2.33	3.78 ± 2.28	1.78e-36
Number of days/week of vigorous physical activity physical activity	1.89 ± 1.96	1.98 ± 1.96	2.0e-20
Vegetables and Fruits/day	7.32 ± 4.09	7.66 ± 3.78	5.32e-63
Fish/week	3.45 ± 1.44	3.67 ± 1.36	2.55e-197
Meat/week	7.79 ± 2.83	7.78 ± 2.62	0.41
Alcohol consumption in g/d	9.07 ± 10.14	8.73 ± 9.63	7.92e-11
Multivitamin intake	2.5	25.4	1.0e-300
Vitamin C intake	0.1	0.8	1.23e-247
Vitamin B12 intake	0.2	0.1	0.06
Townsend Index	−1.40 ± 3.03	−1.81 ± 2.78	1.88e-158

Quantitative values are expressed as mean ± standard deviation. Categorical variables are expressed as relative frequencies in percent (%).

For continuous variables, univariate *p*-values were calculated using an independent t-test. For categorical variables, *p*-values were calculated using chi-square test. Abbreviations used: BMI, body mass index.

We then analyzed the incidence of liver diseases: Omega-3 users had a significantly lower incidence of overall liver disease than non-users (HR = 0.716; 95% CI: 0.639, 0.802; *p* = 7.6 × 10^−9^) (Table 2). In particular, new “alcoholic liver disease” (K70) (HR = 0.559; 95% CI: 0.347, 0.833; *p* = 4.3 × 10^−3^) as well as hepatic failure (HR = 0.548; 95% CI: 0.343, 0.875; *p* = 1.2 × 10^−2^) were significantly reduced in Omega-3 users. In addition, we found a risk reduction for “other diseases of liver” (K76), including NAFLD (HR = 0.784; 95%CI: 0.650, 0.944; *p* = 1.0 × 10^−2^). We replicated this association in a subgroup of UKB patients who underwent MRI. Here, Omega-3 intake was associated with less MRI-confirmed steatosis (HR = 0.846; 95% CI: 0.777, 0.921; *p* = 1.1 × 10^−4^) (Table 2).

**Table 2:** Omega-3 intake and the development of incident liver disease and hepatocellular carcinoma in individuals without prior liver disease in UKB.

	**Omega-3 intake**		**No Omega-3 intake**		
	**No. with event/ total No**.	**Hazard ratio (95% CI)**	**No. with event/ total No**.	**Hazard ratio (95% CI)**	***p*-value**
Incident Liver disease^*^	350/38,121	**0.716 (0.639 to 0.802)**	4,010/373,035	1.00 (reference)	**7.6e-09**
Alcoholic liver disease (K70)	27/38,121	**0.559 (0.347 to 0.833)**	404/373,035	1.00 (reference)	**4.3e-03**
Toxic liver disease (K71)	5/38,121	0.96 (0.36 to 2.58)	34/373,035	1.00 (reference)	0.94
Hepatic failure, not elsewhere classified (K72)	20/38,121	**0.548 (0.343 to 0.875)**	270/373,035	1.00 (reference)	**1.2e-02**
Chronic hepatitis, not elsewhere classified (K73)	7/38,121	1.42 (0.61 to 3.30)	51/373,035	1.00 (reference)	0.42
Fibrosis and cirrhosis of liver (K74)	52/38,121	0.76 (0.57 to 1.02)	558/373,035	1.00 (reference)	0.07
Other inflammatory liver diseases (K75)	61/38,121	1.02 (0.77 to 1.34)	508/373,035	1.00 (reference)	0.90
Other diseases of liver (K76)	253/38,121	**0.711 (0.623 to 0.812)**	3,034/373,035	1.00 (reference)	**5.1e-07**
Fatty (change of) liver, not elsewhere classified (K76.0)	128/38,121	**0.784 (0.650 to 0.944)**	1,533/373,035	1.00 (reference)	**1.0e-02**
MRI confirmed steatosis (>5% liver fat)	832/38,121^**^	**0.846 (0.777 to 0.921)**	7,546/373,035^***^	1.00 (reference)	**1.1e-04**
Liver disorders in diseases classified elsewhere (K77)	<5/38,121	2.00 (0.38 to 10.51)	8/373,035	1.00 (reference)	0.42
Liver cell carcinoma (C220)	9/38,121	0.66 (0.32 to 1.33)	108/373,035	1.00 (reference)	0.24

^*^Incident Liver Disease is defined as new onset Liver Disease K70-K77 after Baseline examination. ^**^34,905 missing values and ^***^345,035 missing values. Bold values are significant (*p*-value < 0.05).

Men with regular Omega-3 intake showed a 27.8% reduction in the risk of a new diagnosis of liver disease (HR = 0.722; 95% CI: 0.611, 0.854; *p* = 1.4 × 10^−4^), whereas women showed a 28.5% reduction (HR = 0.715, 95% CI: 0.613, 0.834; *p* = 2.0 × 10^−5^) (Table 3).

**Table 3:** Associations of Omega-3 intake on the risk of incident liver disease in UKB.

	No. with event/ total No.^*^	**Hazard ratio (95% CI)**	***p-*value**
**Incident liver disease** ^**^			
In Men^***^	157/15,373	**0.722 (0.611 to 0.854)**	**1.4e-04**
In Women^***^	193/22,748	**0.715 (0.613 to 0.834)**	**2.0e-05**
*PNPLA3* rs738409 (wt)	184/23,023	**0.679 (0.580 to 0.793)**	**1.0e-06**
*PNPLA3* rs738409 (het)	122/12,496	**0.729 (0.602 to 0.884)**	**1.0e-03**
*PNPLA3* rs738409 (hom)	33/1,698	0.96 (0.66 to 1.41)	0.84
*TM6SF2* rs58542926 (wt)	285/31,817	**0.722 (0.636 to 0.818)**	**3.8e-07**
*TM6SF2* rs58542926 (het)	53/5,127	0.76 (0.57 to 1.01)	0.06
*TM6SF2* rs58542926 (hom)	<5/213	0.38 (0.08 to 1.74)	0.21
*HSD17B13* rs72613567 (wt)	173/19,536	**0.686 (0.584 to 0.805)**	**4.0e-06**
*HSD17B13* rs72613567 (het)	141/14,849	**0.775 (0.647 to 0.927)**	**5.3e-03**
*HSD17B13* rs72613567 (hom)	23/2,719	**0.610 (0.393 to 0.949)**	**2.8e-02**
*MTARC1* rs2642438 (wt)	165/18,456	**0.710 (0.602 to 0.837)**	**4.6e-05**
*MTARC1* rs2642438 (het)	147/15,407	**0.738 (0.619 to 0.880)**	**7.1e-04**
*MTARC1* rs2642438 (hom)	27/3322	**0.660 (0.439 to 0.992)**	**4.6e-02**

^*^For sensitivity analyses, only individuals taking Omega-3 were referred to, with hazard ratios and *p*-values calculated consistently compared to individuals not taking Omega-3. ^**^Incident Liver Disease is defined as new onset Liver Disease K70-K77 after Baseline examination. ^***^Sex was excluded from the covariates. Bold values are significant (*p*-value < 0.05).

### Multivitamins, vitamin C and vitamin B12 are not associated with risk reduction for incident liver disease

It is important to consider potential confounding factors, such as other supplements or medications, that may have an impact on the outcome being studied, to improve the accuracy of the results and draw more valid causal inferences. Therefore, we studied the intake of multivitamins, vitamin C, or vitamin B12 nutritional supplements in the UKB. Vitamin C and vitamin B12 have shown associations with liver health in previous studies (13–15). Nevertheless, our results showed no significant benefit for incident liver disease (Table 4).

**Table 4:** Intake of different nutritional supplements and the development of incident liver disease in individuals without prior liver disease in UKB.

**Event and treatment group**	**No. with event/ total No**.	**Hazard ratio (95% CI)**	***p*-value**
**Incident liver disease** ^*^			
Multivitamin			
No Multivitamin intake	4,155/392,107	1.00 (reference)	-
Multivitamin intake	205/19,049	0.95 (0.82 to 1.10)	0.47
Vitamin C			
No Vitamin C intake	4,348/410,543	1.00 (reference)	-
Vitamin C intake	12/613	1.45 (0.82 to 2.56)	0.20
Vitamin B12			
No Vitamin B12 intake	4,342/410,447	1.00 (reference)	-
Vitamin B12 intake	18/709	1.21 (0.76 1.92)	0.43

^*^Incident Liver Disease is defined as new onset Liver Disease K70-K77 after Baseline examination.

### Omega-3 intake is protective regardless of genetic risk

Common genetic variants in *PNPLA3* and *TM6SF2* have been shown to increase the risk and severity of NAFLD, whereas single nucleotide polymorphisms (SNPs) in *MTARC1* and *HSD17B13* have protective effects (16–19). Similar to the results in the general population, Omega-3 intake resulted in a 27.1% risk reduction of incident liver disease in heterozygous carriers of the minor allele of *PNPLA3* rs738409 (HR = 0.729; 95% CI: 0.602, 0.884; *p* = 1.0 × 10^−3^) (Table 3). Importantly, this association was not observed in homozygous carriers of the minor allele of *PNPLA3* rs738409. In contrast, minor allele carriers of *TM6SF2* rs58542926 showed no significant risk reduction. We next analyzed whether Omega-3 supplementation has an additive protective effect on common protective genetic variants. Interestingly, in homozygous carriers of the minor allele of *HSD17B13* rs72613567 the protective effect of Omega-3 supplementation was strikingly higher than in the general population (HR = 0.610; 95% CI: 0.393, 0.949; *p* = 2.8 × 10^−2^). Similar results were obtained for *MTARC1* rs2642438, suggesting additive hepatoprotective effects or potential synergistic effects (Table 3). Additionally, we repeated the analyses in the smaller subset of patients with measured Omega-3 levels and found comparable results (Supplementary Table S3).

### Omega-3 intake in individuals with lipidomic data

Finally, we examined the individuals who received an analysis of the lipidomic data and evaluated the differences between Omega-3 users and non-users in more detail (Supplementary Table S3). The incidence of liver disease was significantly lower among Omega-3 supplement users (81 cases/8,747 individuals) compared to non-users (847 cases/82,218 individuals), indicating a potential protective effect of Omega-3 supplementation against liver disease. Overall, Omega-3 intake was significantly associated with a lower risk of liver disease (HR = 0.726; 95% CI: 0.573, 0.921; *p* = 8.0 × 10^−3^). Stratified analysis by gender revealed a significant association in women (HR = 0.653; 95% CI: 0.467, 0.912; *p* = 1.2 × 10^−2^), but not in men.

## Discussion

Dietary modification is a cornerstone in the treatment of NAFLD. However, research on how targeted dietary interventions can be used for primary prevention is limited. This study aimed to investigate the relationship between Omega-3 fatty acid consumption and the development of liver disease in a large and diverse population-based cohort, including both non-alcoholic and alcoholic liver disease. We found that regular Omega-3 fatty acid consumption was associated with a significant risk reduction in liver disease development, particularly for (non-)alcoholic liver disease.

Notably, our investigation included a comprehensive analysis of both general and genetic risk factors for liver disease, and we verified the participants' self-reported Omega-3 intake using lipidomic data.

Our findings indicate that regular Omega-3 fatty acid consumption is associated with a significant reduction in the risk of liver disease, particularly NAFLD. This is consistent with previous studies demonstrating the potential benefits of Omega-3 supplementation in treating NAFLD (5, 8). We further showed that ICD-10 coded NAFLD was significantly reduced in Omega-3 users. Moreover, a significant reduction in the risk of MRI-confirmed steatosis has been observed.

In the UKB, we developed a unique approach to study the interaction between genetics and Omega-3 intake. Especially for heterozygous *PNPLA3* rs738409 minor allele carriers, regular Omega-3 intake showed a benefit. Minor allele carriers of the rare variant *TM6SF2* rs58542926, which is harmful to the liver (17), did not show statistically significant associations, which may have been due to a lack of power. The *HSD17B13* and *MTARC1* variants, which have been linked to lower rates of NAFLD (20, 21), showed opposite associations. Both heterozygous and homozygous minor allele carriers showed a significant risk reduction for *MTARC1* rs2642438 and *HSD17B13* rs72613567. Interestingly, our data indicate an additive synergistic effect of Omega-3 supplementation and protective variants of *HSD17B13* and *MTARC1*. Replication of the results in the subgroup of individuals with lipidomic data confirmed this to a large extent, however, for some genetic variants the number of cases was too small to draw a conclusion. To prevent the lack of significance due to a small sample size, analyzing larger cohorts is recommended, particularly for homozygous minor allele carriers.

One explanation for the hepatoprotective association between Omega-3 intake and liver disease may be that Omega-3 fatty acids improve insulin sensitivity, which is strongly associated with protection against NAFLD (22). In addition, Omega-3 fatty acids may directly affect liver fat metabolism (23). Studies have suggested that Omega-3 fatty acids may increase the breakdown of fat in the liver, leading to a reduction in the accumulation of liver fat, which is consistent with the results of our study (24). Omega-3 fatty acids may also have anti-inflammatory properties, and liver inflammation is a key component in the development and progression of liver diseases (22). The anti-inflammatory, insulin-sensitizing, and lipid-metabolizing effects of Omega-3 fatty acids may contribute to their potential benefits in the prevention of liver diseases, although the exact mechanisms are not fully understood.

This is the largest study to date to demonstrate a primary preventive effect in a prospective and well-characterized cohort. Moreover, gene-dietary interactions have not been studied at a population-based level before and this research may uncover personalized treatment strategies for individuals at risk for metabolic liver diseases. Deciphering gene-environment interactions in metabolic liver disease holds promise for the development of patient-tailored dietary strategies. Nevertheless, our study has limitations. First, we used ICD-10 diagnoses as outcomes in our study, which were continuously updated. In spite of this, we confirmed the negative association between Omega-3 supplementation and hepatic steatosis using MRI data. However, we recommend the replication of genetic findings in a larger cohort. Second, the frequency of Omega-3 intake was not available for analysis except for the statement that it was taken regularly. We attempted to mitigate these limitations by assessing the blood metabolites indicative of Omega-3 use which strongly supports the reported intake (Figure 2, Supplementary Table S2). Furthermore, Omega-3 supplementation may indicate an overall healthier lifestyle, which could not be entirely excluded. To correct for factors associated with a healthier lifestyle, we included pre-existing conditions, physical activity, dietary factors, other vitamin supplements, and socioeconomic status (Table 1). Moreover, we cannot completely exclude the influence of different cooking oils on Omega-3 serum levels by correcting for dietary factors. In addition, the lack of analysis on specific Omega-3 associated fatty acids such as eicosapentaenoic acid (EPA) or docosahexaenoic acid (DHA) is a limitation that should be addressed in cohorts with corresponding data to provide a nuanced understanding of the relationship between Omega-3 fatty acids and liver health. Bias due to misclassification was minimized by sensitivity analyses with different endpoints (Tables 2–4). To further mitigate the risk of residual confounding, we performed sensitivity analyses in subgroups in which the association persisted robustly (Tables 3, 4).

Our study raises the question of whether Omega-3 supplementation should be recommended to people at a high risk of liver disease. Supplementation with Omega-3 fatty acids was particularly beneficial for women. However, these associations need to be confirmed in randomized trials before recommending Omega-3 for protection against liver disease.

In conclusion, this study demonstrated the primary preventive associations of Omega-3 supplementation with the development of incident liver disease, which warrants further evaluation in clinical trials.

## Data availability statement

The data analyzed in this study is subject to the following licenses/restrictions: approved registration at UK Biobank is required. Requests to access these datasets should be directed to https://www.ukbiobank.ac.uk.

## Ethics statement

The studies involving human participants were reviewed and approved by the Northwest Multicenter Research Ethics Committee. The patients/participants provided their written informed consent to participate in this study.

## Author contributions

MV: conceptualization, methodology, software, formal analysis, writing—original draft, and validation. KC and ES: resources, writing—review and editing, and validation. KSS, LH, and MR: writing—review and editing. KMS: conceptualization, writing—review and editing, and funding acquisition. CS: conceptualization, software, writing—review and editing, supervision, funding acquisition, and validation. All authors contributed to the article and approved the submitted version.
