# Supplementary material for: Omega-3 intake is associated with liver disease protection
Source: Front Public Health. 2023 Jul 19;11:1192099. doi: 10.3389/fpubh.2023.1192099 (PMC10394692; doi:10.3389/fpubh.2023.1192099)
Supplement: Supplementary file 1 [file Data_Sheet_1.pdf]

Supplementary Appendix

This supplement was provided by the authors to give further information on the analyses.

Mara Sophie Vell, Kate Townsend Creasy, PhD, Eleonora Scorletti, MD, PhD, Katharina Sophie Seeling, Leonida Hehl, Miriam Daphne Rendel, Kai Markus Schneider, MD PhD, Carolin Victoria Schneider, MD

Supplementary Appendix

Supplementary Tables S1-S3………..Page 3-6

Table S1

**Table S1.** Numerical code of the medication		
**Medication**	**Specification**	**Included Code**
Aspirin*		1140909772, 1140861804, 1140868226, 1140861806, 1140882392, 1140882268, 1140882108, 1140882190, 1140868282, 1140872040, 1141163138, 1141167848, 1140909888, 1140871080, 1140925942, 1140923344, 1140856336, 1141167844, 1140861808, 1140882192, 1141151924, 1140868264, 1140882106, 1140856394, 1141164050, 1141164044, 1140856314, 1140863514, 1140856220, 1141177826, 1140872032, 1140864860, 1140856212, 1141188536, 1140917408, 1140868294, 1140856440, 1140856214, 1140856344
Biguanide*	Metformin	1140921964, 1140874686, 1140884600, 1141189090, 1141153138
Insulin		1140883066
Omega-3		1140861884, 1141181868, 1193, 1140909674
Statin	Atorvastatin, Fluvastatin, Pravastatin, Rosuvastatin, Simvastatin	1141146234, 1141192414, 1140910632, 1140888594, 1140864592, 1141146138, 1140861970, 1140888648, 1141195196, 1141192410, 1141188146, 1140861958, 1140910652, 1140910654, 1140881748, 1141200040
Multivitamin Supplements*		1140870488,1140858368,1140877706,1141164602,1140876592,1140852976,1140909766,1141167678,1140852916,1140852920,1140871060,1140871162,1141173902
Vitamin C Supplement*		1140909730,1140858300,1140858370,1140852900,1140870786,1140852870,1140852872,1140852908,1140870492,1140852910,1140870946,1140911682,1140852918
Vitamin B12 Supplement*		1140870508,1140870512,1140870516,1140870520,1140858304,1140858306,1140858452,1141173348,1140870504,1140852900,1140852870,1140870492,1140852918

*Combined preparations possible. Particularly in the case of combined nutritional supplements, there can be an overlap of numerical codes.

Table S2

**Table S2.** Metabolic profile ω-3 users		
	**beta**	**-log$_{10}$(p-value)**
Phosphoglycerides	0.004	1.607
Sphingomyelins	0.011	10.238
ApoB	0.017	10.723
ApoA1	-0.001	0.157
Total Fatty Acids	0.002	0.374
Omega-3 Fatty Acids	0.125	161.574
Omega-6 Fatty Acids	0.002	0.693
PUFA	0.015	16.439
MUFA	-0.016	5.744
Saturated Fatty Acids	-0.003	0.525
Linoleic Acid	0.000	0.077
Docosahexaenoic Acid	0.115	203.450
Albumin	0.007	10.320
Glycoprotein Acetyls	-0.010	9.137
Concentration of Large VLDL Particles	0.002	0.099
Total Lipids in Large VLDL	0.005	0.407
PL in Large VLDL	-0.004	0.268
C in Large VLDL	0.009	0.999
TG in Large VLDL	0.006	0.528
Concentration of Medium VLDL Particles	0.019	6.076
Total Lipids in Medium VLDL	0.017	4.788
PL in Medium VLDL	0.020	5.641
C in Medium VLDL	0.029	11.189
TG in Medium VLDL	0.009	1.308
Concentration of Small VLDL Particles	0.011	2.562
Total Lipids in Small VLDL	0.012	2.998
PL in Small VLDL	0.017	6.237
C in Small VLDL	0.018	5.922
TG in Small VLDL	0.002	0.234
Concentration of Large LDL Particles	0.017	9.941
Total Lipids in Large LDL	0.021	14.731
PL in Large LDL	0.021	15.576
C in Large LDL	0.023	16.489
TG in Large LDL	-0.006	1.428
Concentration of Medium LDL Particles	0.024	16.157
Total Lipids in Medium LDL	0.022	12.870
PL in Medium LDL	0.021	12.378
C in Medium LDL	0.025	14.908
TG in Medium LDL	-0.007	1.215
Concentration of Small LDL Particles	0.016	10.742
Total Lipids in Small LDL	0.018	12.024
PL in Small LDL	0.018	14.456
C in Small LDL	0.020	13.362
TG in Small LDL	-0.010	1.872
Concentration of Large HDL Particles	0.006	0.641
Total Lipids in Large HDL	0.004	0.364
PL in Large HDL	0.001	0.114
C in Large HDL	0.008	0.880
TG in Large HDL	-0.014	2.682
Concentration of Medium HDL Particles	-0.003	0.543

Total Lipids in Medium HDL	-0.004	1.197
PL in Medium HDL	-0.006	2.173
C in Medium HDL	-0.001	0.129
TG in Medium HDL	-0.019	5.888
Concentration of Small HDL Particles	0.003	1.532
Total Lipids in Small HDL	-0.001	0.268
PL in Small HDL	-0.003	1.418
C in Small HDL	0.004	1.909
TG in Small HDL	-0.013	3.300
Abbreviaitons: C, Cholesterol; TG, Triglycerides; PL, Phospholipids; VLDL, Very-low-density lipoprotein; LDL, Low-density lipoprotein; HDL, High-density lipoprotein; ApoB, Apolipoprotein B; ApoA1, Apolipoprotein A1; PUFA, Polyunsaturated fatty acids; MUFA, Monounsaturated fatty acids.		

Table S3

**Table S3.** Associations of Omega-3 intake in patients with lipiodomic data in UKB			
**Incident Liver disease (K70-K77)**	**No Omega-3 intake (N=82,218)**	**Omega-3 intake (N=8,747)**	
No diagnosis (K70-K77)	81,371/82,218	8666/8747	
Diagnosis (K70-K77)	847/82,218	81/8747	
	**No. with Event/ Total No.***	**Hazard Ratio (95% CI)**	**p-value**
		**Incident Liver Disease****	
**Overall**	81/8747	**0.726 (0.573 to 0.921)**	**8.0e-03**
**in Men*****	40/3527	0.83 (0.60 to 1.17)	0.29
**in Women*****	41/5220	**0.653 (0.467 to 0.912)**	**1.2e-02**
***PNPLA3* rs738409 (wt)**	47/5383	**0.729 (0.533 to 0.997)**	**4.8e-02**
***PNPLA3* rs738409 (het)**	2/2896	0.69 (0.46 to 1.04)	0.08
***PNPLA3* rs738409 (hom)**	7/404	0.98 (0.42 to 2.32)	0.96
***TM6SF2* rs58542926 (wt)**	73/7443	**0.769 (0.598 to 0.989)**	**4.1e-02**
***TM6SF2* rs58542926 (het)**	8/1176	0.59 (0.28 to 1.24)	0.17
***TM6SF2* rs58542926 (hom)**	0/54	-	-
***HSD17B13* rs72613567 (wt)**	38/4598	**0.670 (0.475 to 0.945)**	**2.2e-02**
***HSD17B13* rs72613567 (het)**	38/3443	0.82 (0.58 to 1.17)	0.27
***HSD17B13* rs72613567 (hom)**	4/625	0.54 (0.19 to 1.54)	0.25
***MTARC1* rs2642438 (wt)**	37/4289	0.75 (0.53 to 1.07)	0.11
***MTARC1* rs2642438 (het)**	38/3637	0.75 (0.53 to 1.07)	0.11
***MTARC1* rs2642438 (hom)**	6/754	0.58 (0.24 to 1.39)	0.23
